# The use of alcohol-free and low-alcohol drinks in pregnancy in the UK

**DOI:** 10.1093/eurpub/ckaf188

**Published:** 2025-11-05

**Authors:** Kate Maslin, Heather Hopper, Jill Shawe

**Affiliations:** School of Nursing and Midwifery, University of Plymouth, Devon, United Kingdom; Department of Development and Regeneration, KU Leuven, Leuven, Belgium; School of Nursing and Midwifery, University of Plymouth, Devon, United Kingdom; Department of Development and Regeneration, KU Leuven, Leuven, Belgium; University Hospitals Plymouth NHS Trust, Devon, United Kingdom; School of Nursing and Midwifery, University of Plymouth, Devon, United Kingdom; Department of Development and Regeneration, KU Leuven, Leuven, Belgium; Royal Cornwall Hospital NHS Trust, Truro, Cornwall, United Kingdom

## Abstract

Alcohol-free drinks [beers, ciders, wines, and spirits containing <0.05% alcohol by volume (ABV)], and low-alcohol drinks (between 0.05% and 1.2% ABV) are increasingly available and may be used as a harm reduction measure. However, it is not known what pregnant women think and feel about these drinks and how regularly they are consumed before and during pregnancy. A cross-sectional online survey was developed and piloted. Women ≥18 years in the UK who were pregnant, or recently pregnant, were recruited via targeted social media advertising. Of the 2092 respondents, 47.8% (*n = *1001) were currently pregnant; 55.7% (*n = *1167) were between 25 and 34 years, 90.0% were White (*n = *1881); 6.1% (*n = *128) were drinking alcohol at “increasing risk” levels (>14 units/week) before pregnancy. During pregnancy, 13.5% (*n = *282) consumed alcohol, which was more common in the increasing risk category (*P < .*01). Alcohol-free or low-alcohol drinks were consumed by 71.3% (*n = *1491) of respondents during pregnancy; 91.4% of the increasing risk category *versus* 69.9% of the lower risk category (*P < .*01). The most common reasons for consuming alcohol-free or low-alcohol drinks were “to choose a safer alternative” (71.9%, *n = *1073) and “to feel included in social events involving alcohol” (68.8%, *n = *1026). More than half of respondents (56.7%) thought there was insufficient information available about consuming alcohol-free and low-alcohol drinks during pregnancy, with internet searching the primary source of information. Although alcohol-free and low-alcohol drinks are commonly consumed during pregnancy, there are some safety concerns. Their role as a harm reduction measure in those who are drinking alcohol at increasing risk levels prepregnancy needs further investigation.

## Introduction

Alcohol-free and low-alcohol drinks are beers, ciders, wines and spirits containing little or no alcohol. Although thresholds differ between countries, in the United Kingdom (UK), alcohol-free drinks are defined as containing <0.05% alcohol by volume (ABV), whereas low-alcohol drinks contain between 0.05% and 1.2% ABV [1]. The consumption of these drinks is a rapidly growing trend, with 9.8% of adults consuming them on a weekly basis in the UK [2], alongside rising consumption across Europe [3]. The increased popularity has led to public health policy interest in whether they could contribute to reduced alcohol intake and associated harms [4], with recent evidence they are increasingly purchased by heavier drinkers [5]. Although it is reported that they are more commonly consumed by men than women (11.1% of men *versus* 8.5% of women) [2], little is known about their use before or during pregnancy.

As alcohol is a known teratogen, the World Health Organization, advise there is no safe level of intake during pregnancy [6]. Despite this widespread advice, it is estimated that ∼9.8% of pregnant women globally and one quarter of pregnant women in Europe consume alcohol [7]. The reasons for this are complex and influenced by a range of sociocultural factors [8]. At 41.3%, the UK has one of the highest rates, translating to one of the highest rates (1.8%–3.6% of school age children) of Foetal Alcohol Spectrum Disorder (FASD) [7], a group of neurodevelopmental conditions associated with poorer physical, mental health and educational outcomes [9]. Alcohol consumption in the preconception phase is less documented, as it is not a routinely collected metric in primary healthcare [10], complicated by the fact that almost half of pregnancies in the UK are unplanned [11].

How patterns of alcohol consumption before and during pregnancy, relate to attitudes to and intake of alcohol-free and low-alcohol drinks is unclear and merits further investigation. This is especially pertinent and timely given the relatively high rates of alcohol intake during pregnancy in the UK, their role as a potential harm reduction tool and the increased availability and popularity of these drinks in the past decade. The aim of this study therefore is to understand the awareness and use of alcohol-free and low-alcohol drinks amongst currently or recently pregnant women. Specifically, we were interested in motivations and barriers to consuming these drinks, whether there was any differing risk perception between different ABV levels, and sources of information used.

## Methods

### Participant recruitment and data collection

The eligibility criteria for the study was: women living in the UK, aged ≥18 years, who are currently pregnant, or have been pregnant in the last year. Additionally, respondents needed to be able to read and understand the information sheet in English and have capacity to consent. Participants were recruited online via targeted paid advertising on social media platforms (Facebook, Facebook Messenger, and Instagram) in February 2025. The advertisement was linked to JISC online survey page, a secure platform for hosting questionnaires and summarizing results (https://www.jisc.ac.uk/online-surveys). Separate advertisements were used, tailored to recruit those who were pregnant and postpartum.

### Study documentation and questionnaire

All documentation (questionnaire, information sheet, consent form, and advertisements) was jointly developed and reviewed by Alcohol Change UK, a national charity focused on reducing alcohol harm, and the research team. The questionnaire was peer reviewed and piloted before use. It had five main parts (see Supplementary File S1):

- pregnancy history,
- intake of alcoholic, low-alcohol- and alcohol-free drinks during pregnancy,
- intake of alcoholic, low-alcohol and alcohol-free drinks before pregnancy,
- attitudes and opinions about alcohol-free and low-alcohol drinks when pregnant, and
- demographic characteristics.

The preconception phase was defined as 3 months before pregnancy, therefore information about prepregnancy alcohol consumption was collected using this timeframe. Alcohol consumption was measured using the alcohol use disorders identification test consumption (AUDIT-C) tool [12], which measures the number and frequency of alcohol units consumed.

The definition of alcohol-free and low-alcohol drinks and ABV levels [1] was repeated throughout the questionnaire for clarity. Questions around barriers and motivations to consume alcohol-free and low-alcohol drinks were derived from previous studies [2, 13], whereby respondents could select as many reasons as applicable to them. Respondents could also provide information via a free text option.

### Data analysis

Data was exported and analysed in SPSS version 28.0. Frequencies were tabulated. Participants’ pattern of alcohol consumption prepregnancy was categorized as “low-risk” (⩽14 units/week) or “increasing-risk” (>14 units/week) [14] adapting the AUDIT-C tool scoring [12]. Categorical responses were compared with chi-square or Fisher’s exact tests. Significance tests were two-tailed and alpha set at 0.05.

### Ethical considerations

Favourable ethical approval was granted by the University of Plymouth Faculty of Health Research Ethics and Integrity Committee (reference 5946). Informed consent was gained from all participants.

## Results

### Demographic characteristics

There were 2092 respondents, see Table 1 for details. The majority lived in England (70.0%, *n = *1462) with 10.7%, 10.0% and 9.3% in Wales, Northern Ireland, and Scotland respectively. The majority of participants (90.0%) were of White ethnicity and 47.8% (*n = *1001) were currently pregnant.

**Table 1.** Participant demographic and pregnancy characteristics

	Subcategory	%	*n*
Age (*n = *2091)	18–24 years	1.5	31
	25–34 years	55.8	1167
	35–44 years	42.5	888
	>45 years	0.2	5
Part of UK (*n = *2088)	Wales	10.7	223
	Scotland	9.3	194
	Northern Ireland	10.0	209
	England	70.0	1462
Part of England (*n = *1460)	London	24.0	351
	North East England	9.4	137
	North West England	7.1	103
	Yorkshire	6.3	92
	East Midlands	8.8	128
	West Midlands	8.9	131
	South East England	12.2	178
	East of England	8.7	127
	South West England	14.6	213
Ethnicity (*n = *2088)	Asian or Asian British	4.6	95
	Black	1.4	29
	Mixed or multiple ethnic groups	3.2	67
	White	90.0	1881
	Other ethnic group	0.8	16
Occupational status (*n = *2090)	Working full time	72.1	1507
	Working part-time (<28 hours/week)	18.9	395
	Full-time university/college student	1.1	23
	Looking after family/home	6.0	127
	Unemployed	1.0	21
	Not working because of sickness or disability	0.8	17
Highest educational qualification (*n = *2088)	Postgraduate degree	43.5	908
	Degree	42.7	892
	A-levels or equivalent	7.5	157
	Further education	4.1	86
	Secondary education	1.9	40
	No educational qualifications	0.2	5
Currently pregnant	Yes	47.8	1001
Trimester (*n = *987)	1st	16.5	163
	2nd	41.3	407
	3rd	42.2	417
Pregnant in past 12 months	Yes	52.1	1091
Given birth in past 12 months	Yes	52.7	1103
Parity	Multiparous	61.0	1283
Fertility treatment (*n = *2089)	Yes	11.5	240

### Prepregnancy behaviours

#### Prepregnancy alcohol intake

In the 3 months before pregnancy, 14.5% (*n = *305) reported not to have consumed any alcohol. The most common pattern of alcohol consumption was two to four times per month (36.9%, *n = *772); 14.7% (*n = *308) consumed alcohol two to three times per week; and 1.8% (*n = *38) consumed alcohol ≥4× per week. Participants who drank alcohol consumed 0–2 units on a typical day (41.0%, *n = *732); 6.2% (*n = *110) consumed >7 units on a typical day.

Using the AUDIT-C scoring system as a guide [12], 6.1% (*n = *128) met the Chief Medical Officer [14] definition of consuming alcohol at “increasing risk” (>14 units/week). Those who consumed ⩽14 units of alcohol per week prepregnancy will be termed the “low-risk” group henceforthwith. There was no significant difference between the low-risk and increasing risk groups in terms of age, location, ethnicity, education, or occupational status.

#### Preparation for pregnancy lifestyle changes

In preparation for pregnancy, the most common behaviour change was to take folic acid (75.6%, *n = *1581), followed by stopping or reducing alcohol intake (41.7%, *n = *873). This did not differ by prepregnancy alcohol consumption levels. Those in their first pregnancy were significantly more likely to reduce their alcohol intake prior to pregnancy than those who had been pregnant before (*P < *.01).

#### Prepregnancy consumption of alcohol-free or low-alcohol drinks

In the 3 months before pregnancy, 40.9% (*n = *857) consumed alcohol-free or low-alcohol drinks; 29.3% (*n = *614) consumed them once per month or less, 10.2% (*n = *213) consumed two to four times per month and a small proportion (1.3%, *n = *28) consumed them two to three times per week.

### During pregnancy

#### Alcohol consumption during pregnancy

Most respondents (86.5%, *n = *1810) reported they did not or do not consume any alcohol during pregnancy. This differed by prepregnancy alcohol intake, 87.6% of those in the low-risk category *versus* 70.3% in the increasing risk category (*P < *.01).

Of those who consumed alcohol during pregnancy, most consumed 0–2 units on a typical day (87.1%, *n = *234), whereas 5.4% (*n = *15) consumed 3–4 units, and 2.6% (*n = *7) consumed >5 units.

#### Consumption of alcohol-free drinks and low-alcohol drinks during pregnancy

When asked about consumption of alcohol-free and low-alcohol drinks, 71.3% (*n = *1491) had consumed them during pregnancy. This differed by prepregnancy alcohol consumption category (69.9% of the low-risk category *vs.* 91.4% in the increasing risk category) (*P < *.01).

**Figure 1.**
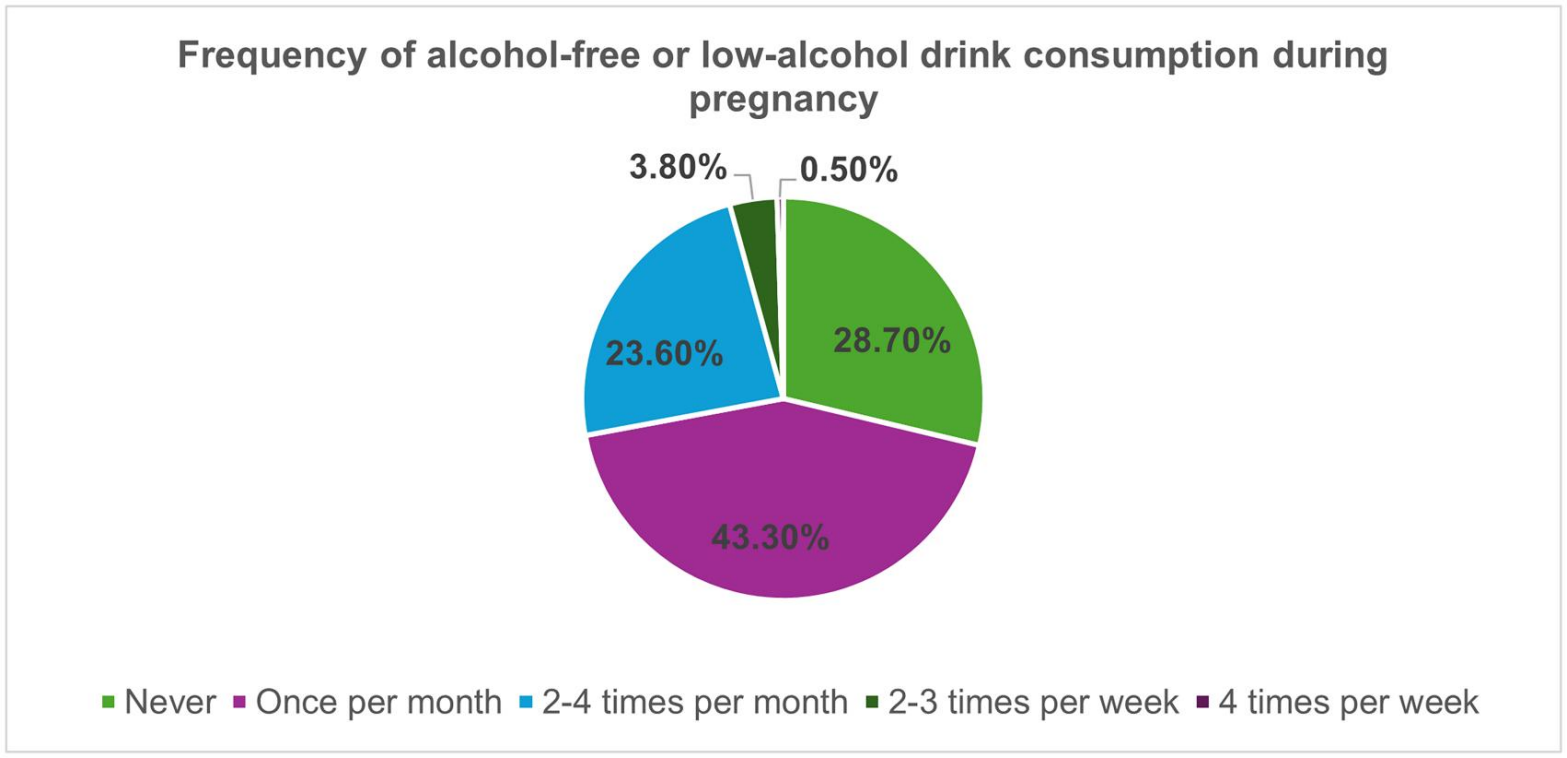
Frequency of consumption of alcohol-free and low-alcohol drinks during pregnancy.

Overall, 43.3%, (*n = *907) drank them once per month or less during pregnancy, 23.6% (*n = *494) drank them two to four times per month, 3.8% (*n = *80) drank them two to three times per week, and 0.5% (*n = *10) drank them more than four times per week (Fig. 1). Those in the prepregnancy increasing risk category were more likely to consume them more frequently, with 46.1% consuming them two to four times per month and 13.3% consuming them two to three times per week (*P < *.01).

#### Reasons for and against consuming alcohol-free and low-alcohol drinks when pregnant

**Figure 2.**
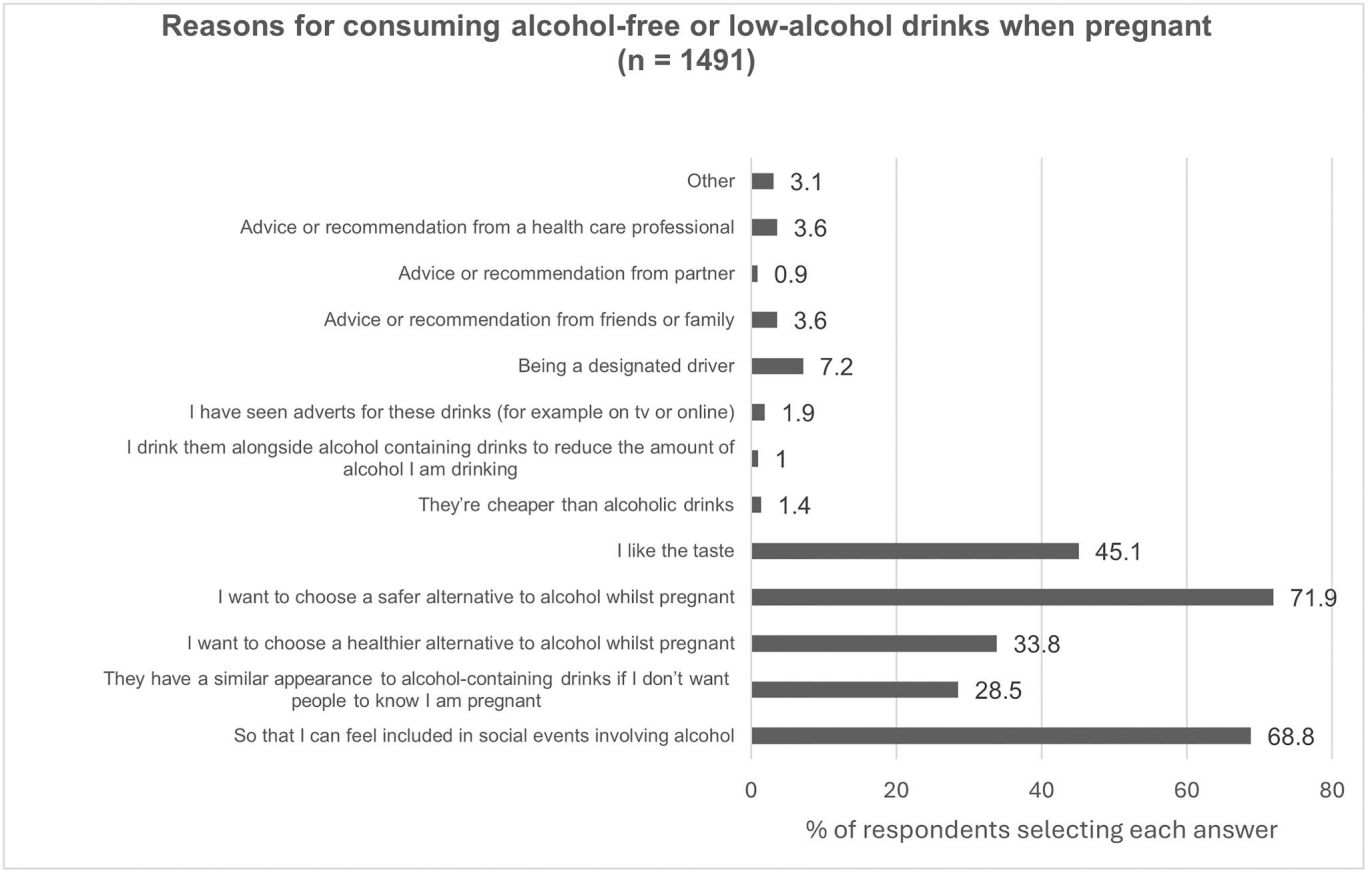
Reasons for consuming alcohol-free or low-alcohol drinks when pregnant.

Participants were asked to select from a list of predefined responses their reasons for consuming alcohol-free and low-alcohol drinks during pregnancy (*n = *1491). The most selected reasons were “to choose a safer alternative to alcohol” (71.9%, *n* = 1073) to “feel included in social events involving alcohol” (68.8%, *n* = 1026), and “I like the taste” (45.2%, *n* = 674) (Fig. 2).

Those who were in the increasing risk group prepregnancy were more likely to choose “feel included in social events involving alcohol” (75.8% *vs*. 47.3% of low risk group, *P* < .001), “they have a similar appearance to alcohol-containing drinks if I don’t want people to know I’m pregnant” (38.3% *vs*. 19.2% of low-risk group *P* < .001), “I want to choose a healthier alternative to alcohol whilst pregnant” (37.5% *vs*. 23.2% of low-risk group, *P* < .001) and “I want to choose a safer alternative to alcohol whilst pregnant” (71.9% *vs*. 50.0%, *P < *.001).

**Figure 3.**
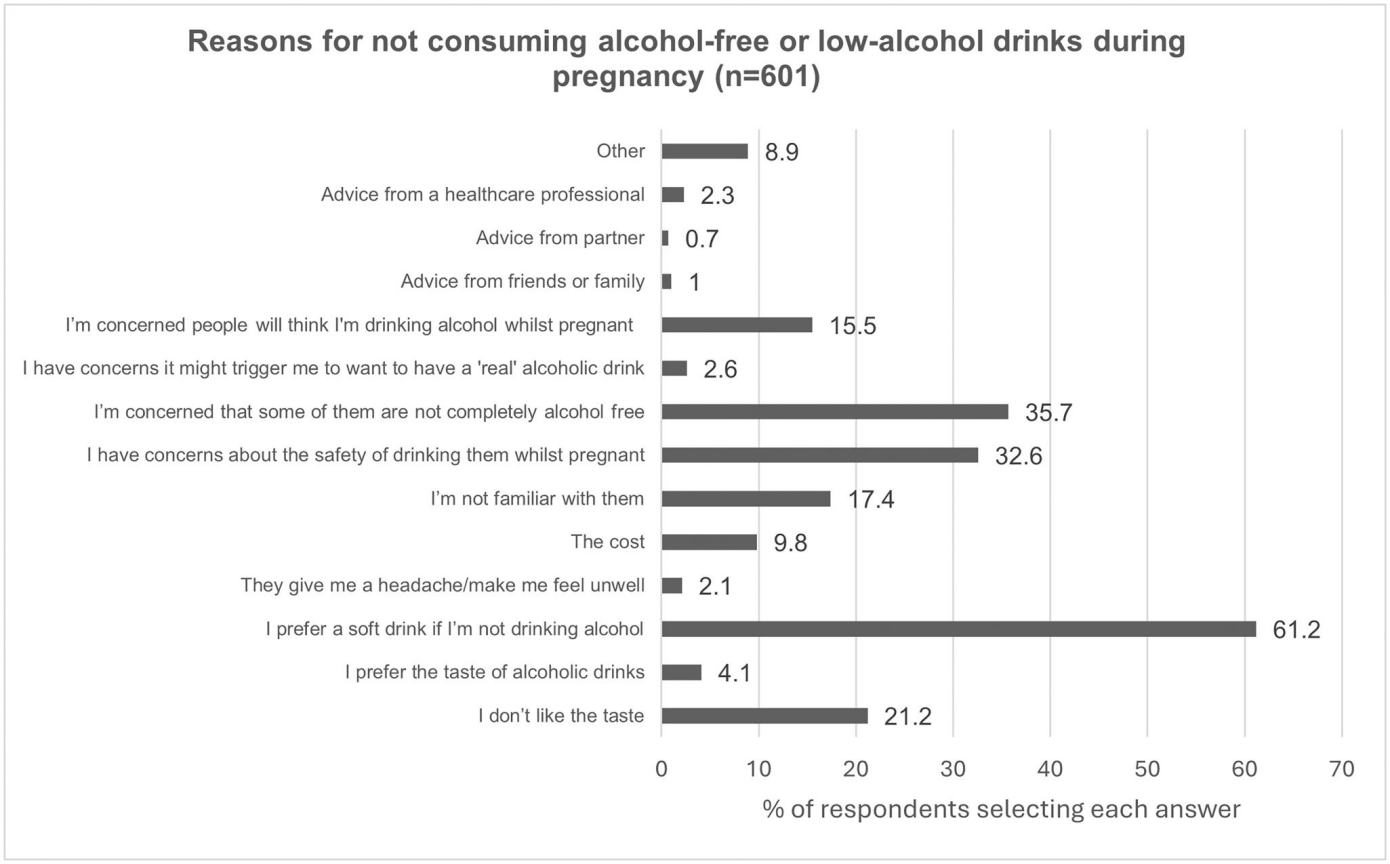
Reasons for not consuming alcohol-free or low-alcohol drinks when pregnant.

When asked to select reasons against consuming alcohol-free or low-alcohol drinks during pregnancy from a list of predefined list responses, there were 601 responses, of which the most commonly selected reasons were “I prefer a soft drink if I’m not drinking alcohol” (61.2%, *n = *368), “I’m concerned that some of them are not completely alcohol free” (35.7%, *n = *215) and “I have concerns about the safety of drinking them whilst pregnant” (32.6%, *n = *196). Further details are shown in Fig. 3.

### Likelihood of consuming alcohol

To gauge whether alcohol-free and low-alcohol drinks were a useful substitute for those who were likely to consume alcohol during pregnancy, participants were asked: *“*If these alcohol-free and low alcohol drinks did not exist, how likely do you think that you would have consumed alcoholic drinks instead? Please answer on a scale of 1–5, where 1 is very unlikely and 5 is very likely”. Overall, 90.6% (*n = *1348) of those who answered this question (*n = *1487), chose 1 (“very unlikely”), with only 1.3% (*n = *19) selecting 5 (“very likely”). This differed by prepregnancy alcohol consumption, with 5.2% of those in the increasing risk category selecting either 4 or 5 (likely to very likely) *versus* 2.2% of the low-risk category selecting this option (*P < *.05).

### Opinions/attitudes about acceptability of consuming different strength drinks during pregnancy

When asked about the acceptability of consuming different strength alcohol drinks during pregnancy on a scale of 1–5, there were varying views. Most respondents (77.8%, *n = *1627) thought consuming alcohol-free drinks (labelled 0% ABV) during pregnancy was very acceptable. This decreased to 10.8% (*n = *225) for low-alcohol drinks (0.5%–1.2% ABV) and to 2.6% (*n = *55) for alcoholic drinks. Those in the “increasing risk” consumption group prepregnancy were more likely to consider that consuming low-alcohol drinks (0.5%–1.2% ABV) during pregnancy were “very acceptable” (20.5% *n = *26, *vs.* 10.2%, *n = *199, *P < *.001).

### Sources of information

More than half of participants (56.7%, *n = *1188) thought there was insufficient information available about drinking alcohol-free and low-alcohol drinks during pregnancy. Participants (18.3%, *n = *383) thought there was enough information and 24.9% (*n = *521) were not sure. This did not differ significantly by prepregnancy alcohol consumption category. When asked if they had received any information about these drinks and pregnancy, more than half of participants had not received information from any of the listed options (54.5%, *n = *1141). Participants (21.6%, *n = *452) had obtained information from self-directed internet searching, 12.9% (*n = *270) from social media, 13.6% (*n = *285) from advertising, and 8.4% (*n = *176) from their midwife.

## Discussion

### Main findings

This UK-based national survey set out to explore and understand the attitudes to and use of alcohol-free and low-alcohol drinks in pregnancy. The survey had excellent participant engagement, with >2000 participants, 6.1% of whom met the definition of consuming alcohol at an “increasing risk” level prepregnancy (>14 units/week) [14]. The proportion of participants who had consumed alcohol-free and/or low-alcohol drinks increased from 40.9% in the 3 months before pregnancy to 71.3% during pregnancy, with more frequent consumption in those in the increasing risk group. Overall, the most selected reasons for consuming alcohol-free or low-alcohol drinks during pregnancy were “to choose a safer alternative to alcohol” (71.9%) to “feel included in social events involving alcohol” (68.8%). Those who were in the increasing risk group prepregnancy were more likely to choose “feel included in social events involving alcohol” (75.8% *vs.* 47.3% of low-risk group) and “they have a similar appearance to alcohol-containing drinks if I don’t want people to know I’m pregnant” (38.3% *vs*. 19.2% of low-risk group). Most respondents (77.8%) thought consuming alcohol-free drinks (labelled 0% ABV) during pregnancy was very acceptable. This decreased to 10.8% for low-alcohol drinks (0.5%–1.2% ABV). More than half of participants (56.7%) thought there was insufficient information available about consuming alcohol-free and low-alcohol drinks during pregnancy, with internet searching the primary source of information.

### What is already known

A major public health concern is the proportion of pregnancies that are unplanned whereby alcohol consumption continues into pregnancy [6]. This is critical as alcohol exposure in the early weeks of pregnancy is highly relevant for risk of FASD. Although there is qualitative evidence that some women find alcohol abstinence guidelines patronizing [15], other research identifies a need for guidance from health care professionals during the transition to motherhood [16]. Research amongst midwives found that although 97% (*n = *842) “always or usually” advised women to abstain from alcohol at their first antenatal appointment, there is no standardized approach to addressing alcohol intake, particularly appointments later in pregnancy [17]. Specifically, the unclear distinction between light drinking and abstinence is a critical challenge for health professionals and pregnant women [18].

In the general population, it is estimated that 15% of adult women in England consume alcohol at increasing risk levels [14], with rates of 9% and 13% for women in the 25–34 and 35–44 year brackets, respectively [19]. Looking more specifically at mothers, an online cross-sectional study (*n = *589) reported of those who drink alcohol, 25.1% were drinking at increasing risk levels [13]. In contrast, it was estimated using a nationally representative sample that 8.5% of women consume alcohol-free and low-alcohol drinks on a weekly basis [2]; however, this was not stratified by age category and no data were collected about pregnancy history or intention. There is potential that alcohol-free and low-alcohol drinks can be a used as harm reduction tool for those who consume alcohol at higher risk levels [5]. However, there is equivocal evidence around an “additionality” effect, by which alcohol-free and low-alcohol drinks are consumed as well as (rather than instead of) alcoholic drinks. A further concern is that due to higher rates of purchasing among more advantaged social groups, alcohol-free and low-alcohol drinks may contribute to the sustaining or widening of health inequalities [2]. Although there is public perception that pregnant women are one of the target groups for products labelled with 0% ABV [20], their use before and during pregnancy is not known.

### What this study adds

To our knowledge, this is the first survey investigating the use of alcohol-free and low-alcohol drinks in those who are pregnant or recently pregnant. The proportion of participants who had consumed alcohol-free and/or low-alcohol drinks increased from 40.9% in the 3 months before pregnancy to 71.3% during pregnancy. Of note, those who were consuming alcohol at increasing risk levels prior to pregnancy consumed alcohol-free or low-alcohol drinks more frequently than those in the low-risk group. This is a considerably higher rate than a 2024 study of the UK public, which reported that 30.2% of women, had consumed alcohol-free or low-alcohol drinks [2].

In terms of reasons for consuming alcohol-free and low-alcohol drinks, the most selected reasons were “to choose a safer alternative to alcohol” (71.9%) to “feel included in social events involving alcohol” (68.8%), similar to findings in the non-pregnant population [21]. A novel finding, specific to the pregnant population was that those who were in the increasing risk prepregnancy were more likely to select “they have a similar appearance to alcohol-containing drinks if I don’t want people to know I’m pregnant” (38.3% *vs*. 19.2% of low-risk group), suggesting that the availability of alcohol-free and low-alcohol drinks could have harm reduction implications for those in early pregnancy. The timing of disclosing pregnancy is a personal choice and due to the risk of miscarriage in the first trimester, many may not wish others to know they are pregnant until at least 12 weeks’ gestation. For those who usually consumed alcohol regularly, but did not wish to make it obvious they were abstaining, alcohol-free or low-alcohol drinks were seen as a useful substitute. Reasons for not consuming these drinks included “I’m concerned that some of them are not completely alcohol free” (35.7%) and “I have concerns about the safety of drinking them whilst pregnant” (32.6%), highlighting concerns about ABV levels and teratogenic risk.

In addition to investigating reasons for and against consuming these drinks during pregnancy, the survey also explored acceptability of different ABV thresholds. Most participants (77.8%, *n = *1627) thought consuming alcohol-free drinks (labelled 0% ABV) during pregnancy was “very acceptable.” This decreased to 10.8% (*n = *225) for low-alcohol drinks (0.5%–1.2% ABV) and to 2.6% (*n = *55) for alcoholic drinks. Linked to perception of safety, participants wanted more information on the suitability of these drinks in pregnancy, with the most common source being self-directed internet searching. Healthcare professionals were not a common source of information, with more respondents sourcing information from advertising (13.6%) than their midwife (8.4%).

### Limitations

As participants were recruited online through social media, there is potential for bias due to digital exclusion and disparity in health literacy. As in most pregnancy research, the sample was skewed towards those of a higher educational level, which is not representative of the UK population and may bias the results. Participants under 18 years were not recruited and only 0.4% of those recruited were >45 years, both age groups at the opposite ends of the reproductive age range, who may have different consumption patterns and attitudes. Despite the strengths of this study, including a large sample size recruited from across the UK, with a representative ethnic mix, findings may not be generalizable to populations outside the UK. Responses were self-reported retrospectively, and due to potential stigma associated with consuming alcohol during pregnancy, intakes may be under reported [22], or subject to recall bias. Specifically, due to the format of the questionnaire, it cannot be determined when alcohol patterns changed, in relation to timing of conception and awareness of pregnancy. Categorization of risk levels was approximated using an adapted version of the AUDIT-C scoring tool [12]. Obstetric outcomes were not captured, as this was outside the scope of the study.

## Conclusion

Although alcohol-free and low-alcohol drinks are commonly consumed during pregnancy, some women are concerned about their safety, with little information received from healthcare professionals. Intake, perception of risk, and acceptability may be influenced by alcohol consumption levels prepregnancy. Clarity is needed on the safety and suitability of alcohol-free and low-alcohol drinks during pregnancy, specifically those in the 0.05%–1.2% ABV category. Clear guidelines about alcohol-free and low-alcohol drinks during pregnancy are needed for health care professionals so that consistent advice can be provided. Their role as a potential harm reduction measure in those who are drinking alcohol at increasing risk levels prepregnancy needs further investigation.

## Supplementary Material

ckaf188_Supplementary_Data

## Data Availability

The data underlying this article will be shared on reasonable request to the corresponding author.
