# Supplementary material for: The use of alcohol-free and low-alcohol drinks in pregnancy in the UK
Source: Eur J Public Health. 2025 Nov 5;35(6):1248–54. doi: 10.1093/eurpub/ckaf188 (PMC12707509; doi:10.1093/eurpub/ckaf188)
Supplement: ckaf188_Supplementary_Data [file ckaf188_supplementary_data.pdf]

Alcohol-free drinks and pregnancy questionnaire

Thank you for agreeing to take part in this study. Please complete the questionnaire as fully as you can. It will take approximately 10 minutes. Please be assured all information collected will be treated confidentially and anonymously.

Please note alcohol-free drinks are defined as beers, ciders, wines, and spirits containing up to 0.05% alcohol by volume (ABV), and low-alcohol drinks as beers, ciders, wines, and spirits containing between 0.05% to 1.2% ABV. Drinks in this category are most commonly 0% and 0.5% ABV. We do not include soft drinks (e.g. fizzy cola, diluting squash, or juice) in this category of drinks.

Part 1: Information about your pregnancy and pregnancy history

1.1 Are you currently pregnant? Yes/No *If yes, continue to Q1.2, if no, proceed to Q1.3*

1.2 Approximately how many weeks pregnant are you? _____ *(proceed to Q3)*

1.3 Have you been pregnant in the last 12 months? Yes/No – *if yes, continue to Q2.1, if no screened out.*

2.1 Have you given birth in the 12 months? Yes/No
(If no, proceed to Q3)

2.2 What type of milk is your baby currently having?
- Breast milk only
- Mixed feeding (infant formula and breast milk)
- Infant formula only
- Not applicable

3. Before you became pregnant, did you do anything to improve your health in preparation for pregnancy? If you have been pregnant more than once, please think of your MOST RECENT pregnancy when answering this question
(Please tick all that apply)
- Took folic acid
- Stopped or cut down smoking
- Stopped or cut down drinking alcohol
- Ate more healthily
- Sought medical/health advice regarding pregnancy
- Took some other action, please describe _________________________________
- I did not do any of the above before my pregnancy

4. Prior to this pregnancy, have you ever been pregnant before? Yes/No (optional)

5. Have you ever undergone fertility treatment? Yes/No (optional)

6. What best describes your future pregnancy plans? *(only for those who are not currently pregnant)*
- Do not want to get pregnant
- Not sure
- Want to get pregnant as soon as possible
- Want to get pregnant in the next 12-18 months
- Want to get pregnant, but no planned timeframe

Part 2: information about your intake of alcoholic, low-alcohol, and alcohol free drinks during pregnancy

7. During your pregnancy, how often do (or did you) have an alcoholic drink?
- Never
- Monthly or less
- 2 to 4 times per month
- 2 to 3 times per week
- 4 times or more per week

If "never" is selected, skip forward to Q9

8.a. During your pregnancy, how many units of alcohol do/did you drink on a typical day when you are drinking?

The picture below shows what a unit is, and how many units are in common drinks.

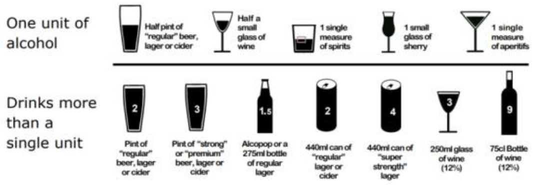

- 0 to 2 units
- 3 to 4 units
- 5 to 6 units
- 7 to 9 units
- 10 or more units

8.b During your pregnancy, which of these alcoholic drinks do/did you drink most often?

- Wine
- Beer or lager
- Spirits on their own (for example whisky, vodka)
- Cider
- Alcopops (for example WKD, Smirnoff Ice)
- Mixed drinks (for example gin and tonic, whisky and coke)
- Other, please state________________

9a. During your pregnancy, how often do you (or did you) drink alcohol-free or low-alcohol drinks (by which we mean drinks that taste like traditional alcohol drinks, but which are 1.2% alcohol or less, most commonly 0% or 0.5% alcohol)?

- Never
- Once per month or less
- 2 to 4 times per month
- 2 to 3 times per week
- 4 times or more per week

If "never" selected skip forward to Q10

9b. During your pregnancy, which of the following alcohol-free or low-alcohol drinks do you (or did you) drink most often?

- Alcohol-free or low-alcohol wine
- Alcohol-free or low-alcohol beer or lager
- Alcohol-free or low-alcohol cider
- Alcohol-free or low-alcohol spirits/cocktails
- Other alcohol-free or low-alcohol drinks, please state____________________

9c. What are/were your reasons for consuming alcohol-free and low-alcohol drinks when pregnant?
Please choose all that apply

- So that I can feel included in social events involving alcohol
- They have a similar appearance to alcohol-containing drinks if I don't want people to know I am pregnant
- I want to choose a healthier alternative to alcohol whilst pregnant
- I want to choose a safer alternative to alcohol whilst pregnant
- I like the taste
- They're cheaper than alcoholic drinks
- I drink them alongside alcohol containing drinks to reduce the amount of alcohol I am drinking
- I have seen adverts for these drinks (for example on tv or online)
- Being a designated driver
- Advice or recommendation from friends or family
- Advice or recommendation from partner
- Advice or recommendation from a health care professional
- Other, please state____________________

(Only visible to those who selected that they do or have consumed them in the past)

9d. If these alcohol-free and low alcohol drinks did not exist, how likely do you think that you would have consumed alcoholic drinks instead? *Please answer on a scale of 1 to 5, where 1 is very unlikely and 5 is very likely*

10. What are/were your reasons for you NOT consuming alcohol free and low-alcohol drinks when pregnant? *Please choose all that apply*

- I don't like the taste
- I prefer the taste of alcoholic drinks
- I prefer a soft drink if I'm not drinking alcohol
- They give me a headache/make me feel unwell
- The cost
- I'm not familiar with them
- I have concerns about the safety of drinking them whilst pregnant
- I'm concerned that some of them are not completely alcohol free
- I have concerns it might trigger me to want to have a 'real' alcoholic drink
- I'm concerned people will think I'm drinking alcohol whilst pregnant
- Advice from friends or family
- Advice from partner
- Advice from a healthcare professional
- Other, please state_______________

<u>Part 3: information about your intake of alcoholic, low-alcohol, and alcohol free drinks BEFORE you became pregnant</u>

11a. Thinking about the 3 months before you became pregnant, how often did have an alcoholic drink?

- Never
- Once per month or less
- to 4 times per month
- to 3 times per week
- times or more per week

If "never" selected skip forward to Q12a

11b. Thinking about the 3 months before you became pregnant, how many units of alcohol did you drink on a typical day when you were drinking?

The picture below shows what a unit is, and how many units are in common drinks.

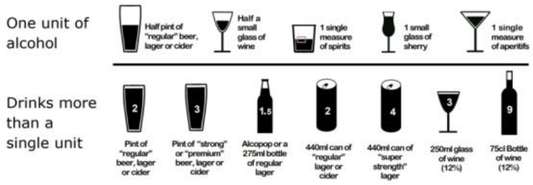

- 0 to 2 units
- to 4 units
- 5 to 6 units
- 7 to 9 units
- 10 or more units

11c Thinking about the 3 months before you became pregnant, which of these alcoholic drinks did you drink most often?

- Wine
- Beer or lager
- Spirits on their own (for example whisky, vodka)
- Cider
- Alcopops (for example WKD, Smirnoff Ice)
- Mixed drinks/cocktails
- Other, please state________________

12a. Thinking about the 3 months before you became pregnant, how often did you drink alcohol-free or low-alcohol drinks?

- Never
- Once per month or less
- 2 to 4 times per month
- 2 to 3 times per week
- 4 times or more per week

If "never" selected skip forward to Part 4

12b. Thinking about the 3 months before you became pregnant, which of the following alcohol-free or low-alcohol drinks did you drink most often?

- Alcohol-free or low-alcohol wine
- Alcohol-free or low-alcohol beer or lager
- Alcohol-free or low-alcohol cider
- Alcohol-free or low-alcohol spirits/cocktails
- Other alcohol-free or low-alcohol drinks, please state____________________

<u>Part 4: Attitudes and opinions about alcohol-free and low-alcohol drinks when pregnant</u>
We would like to understand more about why people do and don't choose to drink alcohol-free and low-alcohol drinks

15. Do you think there is enough information available about the use of alcohol-free and low-alcohol drinks during pregnancy? Yes/No/Not sure

16. Please tick which of the following sources you received information from about alcohol-free and low-alcohol drinks during pregnancy. Please select "none of the above" if you did not receive information from any of these sources

Please *tick all that apply*

- Midwife
- GP
- Other health care professional
- Friend/family member
- Partner
- General internet search, please state any specific website you used_____________
- Social media: please state which platform__________
- Pregnancy organisation or groups, please state__________
- Advertising, please state which brand___________________
- Other, please state__________________
- None of the above

17. Please look at each label below and select whether you think the drink is considered suitable or not suitable to be consumed when pregnant: Yes/No/Not sure

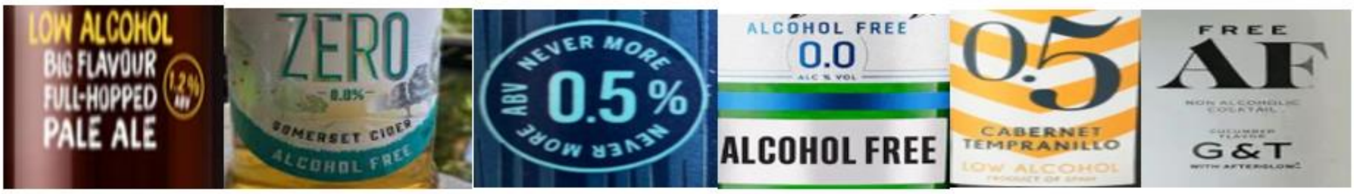

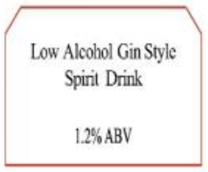

18. To what extent do you think the following are "acceptable" or "unacceptable"?

<u>Please note</u> alcohol-free drinks are defined as beers, ciders, wines, and spirits containing up to 0.05% alcohol by volume (ABV), and low-alcohol drinks as beers, ciders, wines, and spirits containing between 0.05% to 1.2% ABV

Please select on a scale from 1 to 5 where 1 is very unacceptable and 5 is very acceptable:

• Consuming alcohol-free drinks (0% alcohol) during pregnancy
• Consuming low-alcohol drinks (Between 0.5-1.2% ABV) during pregnancy
• Consuming alcoholic drinks during pregnancy

• People who breastfeed consuming alcohol-free drinks (0% alcohol)
• People who breastfeed consuming low-alcohol drinks (Between 0.5-1.2% ABV)
• People who breastfeed consuming alcoholic drinks

19. Finally, please tell us any other thoughts you have around consuming alcohol-free and low-alcohol drinks during pregnancy (and/or breastfeeding) (free text box)

Part 5: For statistical purposes, we would like to know a little more about you: (optional)

20. How old are you? (optional)

- 18-24 years
- 25-34 years
- 35-44 years
- >45 years

21. What part of the UK do you live in? (optional)

- Wales
- Scotland
- England: London/North East/North West/Yorkshire/East Midlands/West Midlands/South East/East of England/South West
- Northern Ireland

22. How would you describe your ethnicity? (optional)

- Asian or Asian British: Indian/Pakistani/Bangladeshi/Chinese/Any other Asian background (please describe________)
- Black: Black British/Caribbean/African/Any other Black, Black British, or Caribbean background (please describe________)
- Mixed or multiple ethnic groups: white and Black Caribbean/White and Black African/White and Asian/Any other mixed or multiple ethnic background (please describe_________)
- White: English, Welsh, Scottish, Northern Irish or British/Irish/Gypsy or Irish Traveller/Roma/Any other White background
- Other ethnic group: Arab, any other ethnic group (please describe____________)

23. What is your highest educational level? (optional)
    - No educational qualifications
    - GCSEs
    - NVQ level 4-5/HNC/HND/RSA Higher diploma/BTEC Higher level
    - A-levels or equivalent
    - Degree
    - Postgraduate qualification

24. What is your main occupational status? (optional) *Please tick the answer that most applies to you (if you're on maternity leave, please tick the answer that applied to you before you had your baby):*
    - Working full time
    - Working part-time (<28 hours/week)
    - Full time university/college student
    - Looking after family/home
    - Unemployed
    - Not working because of sickness or disability
